# Molecular Identification of the Italian Soldiers Found in the Second World War Mass Grave of Ossero

**DOI:** 10.3390/genes16030326

**Published:** 2025-03-11

**Authors:** Barbara Di Stefano, Barbara Bertoglio, Filomena Melchionda, Monica Concato, Solange Sorçaburu Ciglieri, Alessandro Bosetti, Pierangela Grignani, Eros Azzalini, Yasmine Addoum, Raffaella Vetrini, Fabiano Gentile, Francesco Introna, Serena Bonin, Chiara Turchi, Carlo Previderè, Paolo Fattorini

**Affiliations:** 1 Department of Medicine, Surgery and Health, University of Trieste, 34149 Trieste, Italy; barbara.distefano@phd.units.it (B.D.S.); monica.concato@phd.units.it (M.C.); solange.sorcaburuciglieri@units.it (S.S.C.); eazzalini@units.it (E.A.); yasmine.addoum@studenti.units.it (Y.A.); raffaella.vetrini@studenti.units.it (R.V.); sbonin@units.it (S.B.); fattorin@units.it (P.F.); 2 Department of Public Health, Experimental and Forensic Medicine, Section of Legal Medicine and Forensic Sciences, University of Pavia, 27100 Pavia, Italy; barbara.bertoglio@unipv.it (B.B.); pierangela.grignani@unipv.it (P.G.); 3 Section of Legal Medicine, Department of Biomedical Sciences and Public Health, Polytechnic University of Marche, 60126 Ancona, Italy; f.melchionda@staff.univpm.it (F.M.); c.turchi@staff.univpm.it (C.T.); 4 Promega Italia, 20126 Milano, Italy; alessandro.bosetti@promega.com; 5 Reparto Carabinieri Investigazioni Scientifiche di Parma, Sezione di Biologia, 43121 Parma, Italy; fabiano.gentile@carabinieri.it; 6 Section of Legal Medicine, Interdisciplinary Department of Medicine (DIM), University-Hospital of Bari, Giulio Cesare Square 11, 70124 Bari, Italy; francesco.introna@uniba.it

**Keywords:** disaster victim identification, skeletal remains, bones, mass grave, PCR-MPS

## Abstract

Background/objectives: DNA analysis is the most reliable method for the identification of human skeletal remains, especially the ones found in mass disasters or recovered from mass graves. To this aim, DNA was extracted from bones and teeth allegedly belonging to 27 Italian soldiers executed during the Second World War and exhumed from a mass grave in Ossero (Croatia). Methods: A selection of 131 different bone samples (petrous bones, femurs, metacarpal, and metatarsal bones) and 16 molar teeth were used for DNA extraction. Autosomal and Y-chromosome STR profiles were determined using a conventional CE approach, while a panel of 76 microhaplotypes was investigated through MPS. Results: Overall, 24 different autosomal consensus male profiles and six (unexpected) female profiles were identified; the male profiles were then compared with 21 alleged living relatives of the missing soldiers belonging to 14 unrelated Italian families. The DVI module of the Familias software was used for computing the LRs and the posterior probabilities (PP). The combination of autosomal STRs and microhaplotypes led to the identification of six victims and to a very likely identification of another one, supported by Y-haplotype sharing between victim and relative. Three distant victim–relative relationships resulting in low LR values for the autosomal markers showed Y-STR haplotype-sharing patterns, thus suggesting very strong support for a paternal relationship. Conclusions: The results of this study confirmed the effectiveness of the genetic approach and highlighted the presence of more individuals than expected in the mass grave, among which six were female subjects.

## 1. Introduction

Disaster victim identification (DVI) is widely regarded as a key element of humanitarian forensic action (HFA), emphasizing the role of forensic sciences in post-disaster and conflict contexts. The objective of employing forensic medicine and forensic genetics in DVI cases, in either post-human-made or post-natural disasters, is to restore the identities of deceased individuals, addressing and resolving the tragedy of missing persons [1]. The World Trade Center disaster in 2001, caused by an airplane attack [2], and the Southeast Asian tsunami in 2004 [3] represent explicit examples of DVI cases in which many people needed to be identified. Another worrying emergence is also the identification of dead migrants in the Mediterranean Sea, drowned in their desperate attempt to reach Europe [4].

Continuous advancements in forensic genetics have, in fact, allowed DNA profiling to be applied in increasingly complex scenarios such as in the aftermath of mass fatalities and conflicts [5] or in the identification of victims exhumed from dated mass graves [6]. In Europe, for instance, where mass graves are a legacy of various armed conflicts of the 20th century, the exhumation and identification process conducted after the 1992–1995 conflict in Bosnia and Herzegovina shows these efforts, with over 20,000 victims exhumed from several mass graves by the end of 2008 [7,8]. Also, human skeletal remains from mass graves of the Spanish Civil War (1936–1939) have been exhumed and identified [9,10,11]. Similar efforts have led to the genetic identification of skeletal remains from World War II mass graves in Slovenia, Croatia, Poland, Bosnia and Herzegovina, and France [12,13,14,15,16]. The universal right to post-mortem identification is enshrined in domestic and international law [17,18].

The DVI process has been developed over several decades and formalized by developing an internationally recognized sequence of activities. In 1984, the INTERPOL introduced the first DVI guidelines which are regularly updated by a dedicated DVI working group in consultation with four specialized scientific sub-working groups, which are aligned with the key forensic disciplines: odontology, pathology/anthropology, fingerprint analysis, and genetic profiling [19]. This provides forensic science with several tools to assist with the identification of human remains and brings DNA analysis to become one of the irreplaceable ones. Presently, in fact, genetic typing is considered a landmark for individual identification of skeletal remains [5,20]. The procedure relies on the comparison of genetic profiles yielded from post-mortem (PM) samples collected from the deceased with ante-mortem (AM) samples, usually provided by the missing person’s relative/s.

Kinship analysis has a fundamental role in providing invaluable clues for the identification of missing people, disaster victims, and the search for their unknown relatives. The gold standard approach to testing biological relationships between individuals is PCR amplification of sets of STR (short tandem repeat) markers coupled with capillary electrophoresis (STR-CE) separation of the molecular products [21]. As a general rule, the effectiveness of STR markers to infer kinship depends on the number of STRs analyzed and on the availability of a suitable reference database (that is, the ante-mortem database); however, the more distant the relatives, the lower the chances of yielding conclusive results [22]. In such cases, the availability of the recently developed massively parallel sequencing (MPS) platforms has been shown to be a pivotal tool, allowing the analysis of thousands of single nucleotide polymorphisms (SNPs) in a single run [23,24]. Among them, the combination of two–five SNPs along a DNA stretch of less than 300 bp is particularly interesting [25,26]. Such combinations of SNPs have been termed by Kidd [27] as microhaplotypes (microhaps or MHs), and represent a promising tool in forensic genetics because of the low mutation rates, small amplicon size, absence of stutter artefacts, and same-size alleles within any one locus [28,29].

The results of the present report aim for the personal identification of the skeletal remains allegedly belonging to Italian soldiers killed during the Second World War (WWII) and exhumed from a mass grave in Ossero (Croatia). During WWII, in fact, more than 18,500 Italian soldiers were killed or reported missing in the north Balkan region [30]. Around 30 soldiers stationed on the islands of Cres and Lošinj (Kingdom of Italy, at that time) were killed by Tito’s forces at the end of April 1945 and buried in a mass grave close to the Cemetery of Ossero. Official documents dated 12 March 1942 listed the names of 27 Italian soldiers who, having been in those war zones, were believed to have been buried there. As a result, after the excavations of the burial site (May 2019), the skeletal remains were placed in 27 metallic caskets, and transferred to the War Memorial of the Overseas Fallen Soldiers in Bari (Italy).

To identify the deceased soldiers, a kinship analysis was conducted by combining traditional STR-CE methods with the use of a 76 MH-MPS panel as an additional tool to assess LR (likelihood ratio) values. Furthermore, to improve the discrimination power, the possibility of combining the informativity of STR and MH markers was evaluated. Also, paternal lineages were investigated through Y-chromosome STR analysis.

## 2. Materials and Methods

### 2.1. Post-Mortem STR Database Set-Up

The post-mortem STR database, containing the genotypes yielded from the skeletal remains, was built according to the following steps.

#### 2.1.1. Bone/Tooth Samples

In 2019, human remains were uncovered from a mass grave in Ossero (Island of Cres, Croatia). Full details on the mass grave are reported elsewhere [31]. Briefly, historical records suggested that the unearthed remains could belong to 27 Italian soldiers executed on 21 April 1945. The remains were placed in 27 caskets, and delivered to the Italian Government on 13 November 2019 [31]. Later, in 2022, the caskets were transferred to the University of Bari (Italy) for anthropological and medico-legal examinations. These analyses showed a large extent of peri- and post-mortem fractures, with a significant commingling of remains across the 27 caskets. The most likely number of individuals (MLNI) was calculated with standard methods [32] using long bones (femurs, tibiae, and humeri) and skulls [32], revealing an MLNI of 32. In addition, the examination suggested the presence of at least three women (manuscript in preparation).

A total of 341 bone and tooth specimens were sampled and delivered to the University of Trieste (Italy), where they were stored at room temperature in the dark until genetic analysis. According to the literature data [12,13,33,34,35,36,37,38], bone/tooth elements with—a priori—higher chances of being successfully typed were selected. In addition, at least two different bone elements for each of the 27 boxes were considered.

In detail, the following samples were employed: the inner ear portion of the petrous bones [33,34], the cortical of the femur diaphysis [12,13,35], the compact part of the epiphysis of metacarpals and metatarsals [36], and molar teeth (upper right M2) with enamel removed [37,38]. In total, DNA extraction and subsequent molecular analyses were performed on 147 specimens, as shown in Table 1.

#### 2.1.2. Bone/Tooth Cleaning and Pulverization

Bones/teeth were cleaned mechanically using brushes and rotary sanding to remove surface soil. Approximately 1.5 g of bone fragments and whole molar teeth (enamel removed) were treated with 0.5% bleach for 4 min to eliminate external DNA contamination, followed by three washes with sterile bi-distilled water. Surfaces were exposed to UV radiation for 5 min on each side and dried at room temperature overnight [31].

Pulverization was performed using an MM 400 Planetary Ball Mills instrument (Retsch, Haan, Germany) with 25 mL metal grinding vials and 16 mm diameter metal balls (Verder, Vleuten, The Netherlands) at 30 Hz for 1–2 min, using liquid nitrogen to prevent heating [31,39]. The powder samples were stored at room temperature in the dark until use. All procedures were carried out in a room dedicated only to aged bone/tooth handling [31,39].

#### 2.1.3. Bone/Tooth Decalcification and DNA Extraction

Approximately 0.5 g of bone/tooth powder was processed according to the extraction method previously described in detail by Di Stefano et al. [31], which involved overnight decalcification in 0.5 M Na_2_EDTA (pH 8.0), lysis in 460 μL of extraction buffer (1.2% SDS, 10 mM Tris pH 8.0, 10 mM Na_2_EDTA pH 8.0, and 100 mM NaCl), 40 μL of 1M DTT, 80 μL of Proteinase K (20 mg/mL), and automated purification using the Maxwell^®^ FSC DNA IQ^TM^ Casework Kit (Promega) with the Maxwell^®^ RCS TM441 apparatus. The extraction process was conducted on 179 bone/tooth powders (from the 147 bone samples from Table 1; also see Table S1), with a negative extraction control (NEC) included for every 6 samples processed.

#### 2.1.4. DNA Quantification

DNA samples were quantified in duplicate by qPCR using two different kits.

In the first phase of the analyses, the Quantifiler^TM^ Human DNA Quantification Kit (Thermo Fisher Scientific, TFS, Waltham, MA, USA) was used for 107 samples; the CFX96 Real-Time System Instrument (Bio-Rad, Hercules, CA, USA) apparatus was employed following the conditions specified in ref. [40]. This kit targets a 62 bp sequence of the human telomerase reverse transcriptase (hTERT) gene, detected using a FAM-labeled probe; the presence of Taq polymerase inhibitors is monitored using an Internal Positive Control (IPC), labeled by VIC. The Limit of Detection (LOD) was set previously at 0.001 ng/μL [40], while the Limit of Quantification (LOQ) ranged from 0.023 ng/μL to 50 ng/μL. Raw data were analyzed using the CFX Maestro software v5.3.022.1030. (https://www.bio-rad.com; accessed on 13 December 2024).

The remaining 72 samples were quantified using the PowerQuant^TM^ System kit (Promega, Madison, WI, USA) with the 7500 Real-Time PCR System for Human Identification, HID Real-time PCR analysis (Applied Biosystem, AB, Foster City, CA, USA). The kit amplifies an 84 bp short autosomal target and a 294 bp long autosomal target, with the ratio between these two [Auto/Deg] used to assess DNA degradation. It also amplifies a 134 bp target on the Y-chromosome to detect male DNA; an IPC (Internal Positive Control) is added to the reaction to monitor for potential inhibitors. The LOQ for the Auto and Y targets ranged from 0.0032 ng/μL to 50 ng/μL. For the Deg target, the LOQ ranged from 0.0005 ng/μL to 50 ng/μL. The LOD was set at 0.0001 ng/μL for all targets [41]. Raw data analysis was performed using PowerQuant^®^ Analysis Software v2.06 (available on www.promega.com; accessed on 8 January 2025).

#### 2.1.5. STR-CE Typing

As a selection criterion, all the right femurs and the right petrous bone available (for those samples, both autosomal and Y-specific STR panels were used) were analyzed first. With the implementation of the post-mortem autosomal STR database (see Section 2.1.7), the amplification of the Y-specific STR markers was carried out only when a new autosomal profile belonging to a male subject was identified.

The amplification was performed only for samples that showed detectable amounts of DNA in at least one qPCR replicate. So, out of the 179 extracted samples, 126 underwent STR-PCR amplifications (see Table S2).

For autosomal STR amplification, the kits PowerPlex Fusion kit (Promega), PowerPlex ESX17 (Promega), and PowerPlex ESI (Promega) were employed. These kits allow the simultaneous amplification of 22, 17, and 17 autosomal STR markers (plus XY-specific amelogenin targets) in different multiplex configurations, respectively. Y-STR amplification was performed using the PowerPlex Y23 kit (Promega), which amplifies 23 Y-chromosome-specific loci. PCR amplifications were performed under standard conditions for samples containing 0.5–1 ng of DNA template. For samples with a lower DNA quantity, the number of PCR cycles was increased to 32 cycles, and the maximum allowed volume of DNA sample was loaded (17.5 μL for the kits ESX, ESI, and Y23 and 15 μL for the kit Fusion). Positive and negative PCR controls, as well as negative extraction controls (using the maximum allowed volume), were simultaneously run. Amplifications were performed using the T100 Thermal Cycler (Bio-Rad) apparatus.

Capillary electrophoresis was initially carried out using the ABI 310 automatic DNA sequencer and later using the SeqStudio Genetic Analyzer (both from Applied Biosystems, Waltham, MA, USA). Raw data were analyzed with GeneMapperID^®^ ver 3.2.1 software for ABI310 and GeneMapper^TM^ ID X^®^ Software v1.6 (Applied Biosystem) for SeqStudio. The analytical threshold was set at 50 RFU for ABI 310 and 150 RFU for SeqStudio, whereas the stochastic threshold was 150 RFU for ABI 310 and 300 RFU for SeqStudio.

The criterion for including genetic profiles in the post-mortem database was the reliable detection of at least 12 autosomal markers, in accordance with international standards [21,42]. Samples meeting this criterion were amplified in duplicates to achieve consensus [43] profiles; replicate tests were not performed for samples whose profile was already present in the STR autosomal database (that is, the post-mortem database; see Section 2.1.7). The data of replicate amplifications were also used for the assessment of stochastic events, such as allelic drop-in and allelic drop-out phenomena [20,23,44].

#### 2.1.6. Exclusion Database

DNA from the personnel involved in the genetic analysis was typed, and the resulting genotypes were compared with those of the samples under investigation to exclude the possibility of contamination during the procedure [20,23]. In total, five female and four male operators were typed with the STR kits described above.

#### 2.1.7. Post-Mortem Database

The post-mortem database was built to store genotypes yielded from bone/tooth samples. Whenever a suitable post-mortem profile was obtained, it was compared with those already stored in the database to determine if it matched an existing profile or represented a new one. Two different Excel files were created, one for the autosomal and one for the Y-specific STR profiles.

### 2.2. Ante-Mortem Database Set-Up

As the official documents dated 12 March 1942 listed the names of 27 soldiers who could have been buried in the mass grave of Ossero, those data enabled the search for their relatives. Since the descendants/relatives of only 14 missing soldiers requested genetic comparison, buccal swabs of 21 living subjects were collected in total (see Table 2).

As shown in Table 2 and Table S3, the ante-mortem samples included 19 relatives (from 1st to 4th degree) as well as 2 descendants’ mothers (Fam7 and Fam8). Eight male reference samples were connected through the paternal line.

The saliva swabs were shipped by ordinary mail to the Institute of Legal Medicine (Trieste), where they were stored at −20 °C until use. DNA extraction, quantification, and typing were performed following standard methods [20]. Thus, a reference database was created containing the STR profiles of all relatives and the haplotypes of the eight paternally related male individuals.

### 2.3. Preparation of Microhaplotype Libraries and MPS Analysis

This analysis was conducted on a set of families for which the STR-based approach gave inconclusive results (see Section 2.4). PCR primers for MPS libraries were designed on the Ion AmpliSeq Designer tool (Thermo Fisher Scientific, https://ampliseq.com/; accessed on 31 May 2023), keeping the amplicon size below 140 bp to allow even the amplification of degraded samples. The designed MPS panel comprised 76 MH loci with an average effective number of alleles (Ae) value equal to 3.574 and random match probability (RMP) value equal to 1.77 × 10^–66^ in the Italian population (manuscript in preparation).

Libraries were manually prepared in half-reaction volume using the Precision ID Library kit (Thermo Fisher Scientific) according to the manufacturer’s protocol (MAN0017767, rev C.0). Amplifications were performed with DNA input ranging from 1 ng to 90 pg. After partial primer digestion and ligation steps, each library was purified with Agencourt^TM^ AMPure^TM^ XP Reagent (Beckman Coulter, Brea, CA, USA) and finally quantified using the Ion Library TaqMan^®^ Quantification kit (Thermo Fisher Scientific) following the manufacturer’s protocols. The seventeen quantified libraries were diluted to a final concentration of 30 pM. Emulsion PCR and loading onto the chips were performed with an Ion Chef^TM^ Instrument (Thermo Fisher Scientific) and Ion S5^TM^ Precision ID Chef & Sequencing Kit (Thermo Fisher Scientific). Sequencing was performed on an Ion GeneStudio^TM^ S5 System (Thermo Fisher Scientific) and loaded onto an Ion 520^TM^ or Ion 530^TM^ Chip (Thermo Fisher Scientific).

Haplotypes were called using Torrent Suite Version 5.12.3 software on an S5 Torrent Server VM (Thermo Fisher Scientific), together with HID_Microhaplotype_Research_PluginV1.5 (Thermo Fisher Scientific). The plugin was run with the following default settings: minimum of total read coverage per position = 20; minimum number of allele count to include in report = 5; minimum allele frequency (for heterozygous) = 10; and minimum of allele frequency (for homozygous) = 90. The software Integrative Genomics Viewer (IGV, v.2.8.0) was used to visualize and confirm the haplotypes.

The two samples amplified with a low amount of DNA (<0.1 ng) were replicated to consolidate the genotyping results [24,26], and consensus data were used for comparisons.

### 2.4. Statistical Analyses

#### 2.4.1. Autosomal STRs and Microhaplotypes

The genetic profiles of the victims were compared with those obtained from the putative relatives using the DVI module of the Familias software (Version 3.3.1), www.familias.no; accessed on 13 January 2025 [45,46]. A likelihood ratio (LR) value was computed as the likelihood of a specified relationship compared with the hypothesis of unrelatedness. The LR value was combined with non-genetic information, the prior probability, to compute the posterior probability by applying the Bayes’ theorem. In this study, the AM-driven approach was used, considering the number of victims equal to 27 for prior probability calculations and setting the posterior probability for a positive identification to 99.9% [13,42,47,48].

LRs and posterior probabilities were first calculated separately for autosomal STR and MH markers, and subsequently, the information was combined.

For LR and posterior probability calculations, the allele frequencies of the European (strider.online [49]; accessed on 5 January 2025) and the Italian (unpublished) populations were used for the autosomal STR and microhaplotype markers, respectively.

#### 2.4.2. Y-STRs

Matching haplotypes from the AM–PM comparison were analyzed using the kinship analysis tool in the YHRD database, www.yhrd.org; accessed on 14 January 2025 [50], considering three different reference metapopulations (i.e., Eurasian, European, and Western European). The likelihood ratios of the patrilineal relationship compared to non-relationship were computed using the observed counting method and the one-step mutations per transmission event as the calculation method.

## 3. Results

### 3.1. Quantification Results

Table 3a,b show the qPCR-based quantification results of the 179 DNA samples extracted from the 147 skeletal remains considered (replicate extractions were performed on 21.8% of the bone samples; also see Table S1).

Although detectable levels (>LOD) of genetic material were found in all skeletal elements (from 26.7% of the right femur to 100% of the petrous bone), only the petrous bone provided DNA yields in the dynamic range of quantification (Limit of Quantification) in all qPCR tests; the remaining skeletal elements provided values in the LOQ with lower frequencies. The degradation index (calculated for the 40.2% of the samples, i.e., those analyzed with the PowerQuant kit) ranged from 3.9 (metacarpal) to 28.6 (petrous bone), an outcome which appears to be in agreement with the data reported in the literature on this issue [11,12,13,15,21,51]. IPC Cq values did not highlight the presence of inhibition of the qPCR assays.

Out of the 179 extracted samples, 126 (70.4%) provided values above the Limit of Detection in at least one of the two qPCR tests and were, therefore, suitable for STR amplification (according to the criteria we had fixed).

### 3.2. STR Typing Results

**Figure 1:**
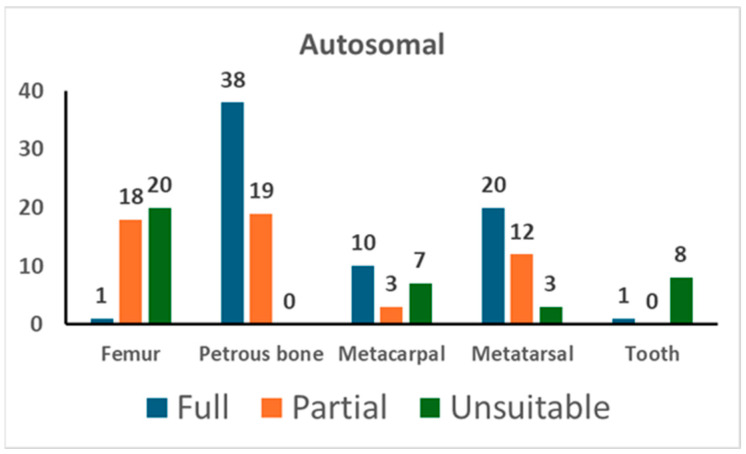
The results of autosomal STR typing. For each skeletal element, the number of full profiles, partial profiles (≥12 markers), and unsuitable profiles is shown (see Section 2 for the criteria assignment).

As shown in Table S2, 126 samples suitable for genetic typing were used for autosomal STR typing in 160 PCR tests. Out of those 160 PCR tests, 122 yielded suitable profiles, that is, full profiles or partial profiles with at least 12 markers (also see Table 4). The results of the autosomal typing are shown in Figure 1.

Amplification of autosomal STR markers yielded full genetic profiles in 66.7% of tests from petrous bone, 57.1% from metatarsals, 50.0% from metacarpals, and 2.6% from femurs. Out of the nine tests conducted on tooth samples, only one provided useful genetic data, whereas unsuitable results were yielded from the remaining eight tests. The partial profiles yielded from petrous bone showed a median of 19 markers. As shown in Table 4, the other skeletal elements yielded partial profiles with a similar number of markers (18 markers in metacarpals, 15 markers in metatarsals, and 18 markers in femurs).

Only for samples yielding a suitable profile for personal identification (≥12 markers) [21,42] were duplicate PCR tests performed. Hence, the cross-checking of the replicates even allowed for the evaluation of stochastic phenomena, such as allelic drop-out and allelic drop-in [20,23,44] (see Table 4). The petrous bone provided replicable genotypes in 96.0% of cases, followed by metatarsals (93.4%), metacarpals (88.1%), and femurs (74.1%). There were no typing data in the blank controls (negative extraction control and PCR-negative control).

**Figure 2:**
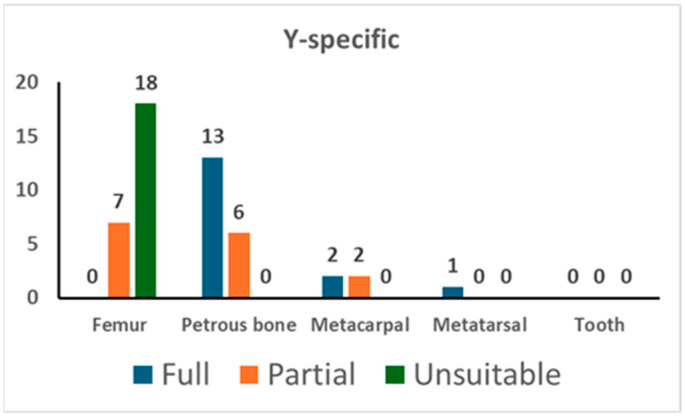
The results of Y-specific STR typing. For each skeletal element, the number of full profiles, partial profiles, and unsuitable profiles is shown (see Section 2 for the criteria assignment).

In addition, the PowerPlex Y-23 kit was used for 49 PCR tests. As shown in Figure 2, 13 out of 19 tests on the petrous bone yielded a full profile while the remaining 6 gave a partial one (with a median value of 20 markers); femurs showed only partial profiles (with a median value of 17 markers) and a high percentage (72.0%) of unsuitable profiles. The analysis of the short bones yielded successful typing in five tests out of five. There were no typing data in the blank controls (NEC and PCR-negative control).

### 3.3. STR Post-Mortem Database

**Figure 3:**
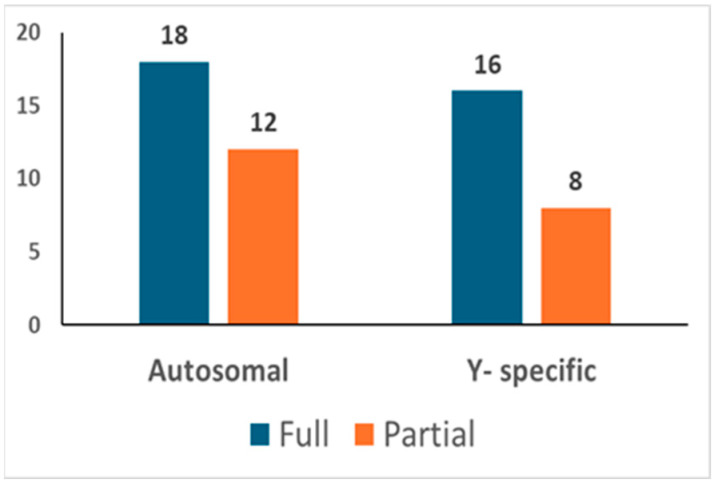
Post-mortem database. The number of autosomal and Y-chromosome consensus profiles (haplotypes) loaded into the post-mortem database.

In total, 92 different bone/tooth samples yielded genetic data potentially suitable for building the post-mortem database (see Table S2). In addition, since none of the 92 profiles matched the profiles of the exclusion database, all 92 were loaded into the database. The inter-sample comparison allowed the identification of 30 unique autosomal STR profiles (see Figure 3). Out of these, 18 were full profiles, whereas 12 were partial profiles (with a median of 15 markers). Since 6 profiles were proven to belong to 6 different female individuals, 24 different Y-STR consensus haplotypes were consequently recorded (16 full and 8 partials with a median of 19 markers). The main features of the six female genotypes found are described in Section 3.3.1.

Figure S1 details the bone/tooth elements, which allowed the definition of the 30 profiles. As shown, eight different bone/tooth samples provided the same STR profile, thus allowing the definition of genotype #2, whereas six genotypes (namely genotypes #22, #25, #26, #28, #29, and #30) were characterized by only one bone sample. On average, three different skeletal elements contributed to the identification of each of the 30 genotypes. The petrous bone contributed to the identification of the genotype in 24 out of 30 cases.

In addition, genetic typing showed that at least 20 caskets contained mixed remains. This outcome is in agreement with the data of an anthropometric analysis which described the remains as highly commingled across the 27 original caskets (manuscript in preparation). Figure S2 shows the trend of the implementation of the post-mortem database, which was constructed from February 2023 to May 2024. Even here, the results clearly show that petrous bone analysis played a determinant role.

#### 3.3.1. Female DNA Profiles

As stated above, six different female genotypes were also scored (see Table S3). In total, female genotypes were identified in eleven different bone elements, which allowed us to define six unique female genotypes (also see Figure S1). The criteria for gender assignment were the lack of amplification of two multicopy male-specific targets (amplicons of 81 bp and 136 bp) in duplicate qPCR analyses with the PowerQuant kit, and the lack of amplification of the male-specific amelogenin target (93 bp) in replicate PCR analyses with the PowerPlex ESI/ESX kits. Out of the six genotypes, two were full (17/17 markers) whereas the remaining four showed partial profiles (with a median value of 15 markers/genotype).

### 3.4. Ante-Mortem Reference Database

As shown in Table S4, relatives of 14 missing soldiers gave their availability for genetic typing. All 21 samples (buccal swabs) used as reference samples yielded full profiles, so they were used to build the ante-mortem reference database.

### 3.5. Kinship Analyses

#### 3.5.1. Autosomal STRs

Family pedigrees were built for the 21 reference persons and were paired with the 24 male victims using the software Familias (ver. 3.3.1). The LR value was calculated considering any of the victims to the missing person compared to an unknown person and posterior probability was computed considering a number of victims equal to 27, using the AM-driven approach provided in the software.

Posterior probabilities, equal to or greater than 99.9%, were obtained in three of the four first-degree cases (Fam01, Fam06, and Fam10) and in Fam07 (99.93%, uncle–niece relationship), while a value very close to 99.9% was achieved in Fam05 (99.59%, full-sibling relationship). The next highest values were observed in Fam03 (96.98%, uncle–nephew relationship) and Fam12 (82.76%, grand-father–grand-nephew relationship), while the remaining seven family groups showed values lower than 35%. The results are summarized in Table 5.

#### 3.5.2. Y-STRs

To detect further possible matches, a patrilinear-driven search was set up by including 23 Y-chromosome STR markers. This analysis was carried out only for the reference persons/victims who were not linked to any victim/family pedigree with posterior probabilities greater than 99.9%, and for whom a patrilineal relationship between the reference person and the missing was stated.

Seven reference persons were thus paired with the twenty victims, and the number of shared alleles was scored. Four haplotype pairs were detected with no exclusion (Fam02, Fam04, Fam08, and Fam09), while one pair (Fam03) showed a single inconsistency at the DYS437 locus, for which a single-step germline mutation was supposed (allele 14 in the victim (uncle) and allele 15 in the reference sample (nephew)). No match was obtained for Fam13 and Fam14. The Y-chromosome haplotypes were searched in the YHRD database (last access: 16 December 2024), and each one was unique in the entire database when the complete set of markers was considered.

Likelihood ratios were computed for each haplotype pair using the kinship analysis tool provided in the database, based on 23 Y-STRs and the Eurasian, European, and Western European reference metapopulations. Since the order of magnitude was the same for the three reference populations, the match was summarized by reporting the most conservative LR value corresponding to the Western European metapopulation (see Table 5). LRs equal to 1 × 10^4^ were obtained for all the matching pairs, with the exception of Fam03, for which an LR equal to 1 × 10^1^ was computed because of the inconsistency at the DYS437 locus.

### 3.6. Microhaplotype Data and Genotyping

This analysis was conducted on five victim–reference person pairs showing posterior probabilities lower than 99.9% and sharing the same Y-STR haplotype. Fam05 and Fam12 were included for the high posterior value obtained with autosomal STRs (99.59% and 82.76%, respectively). Overall, a selection of 7 familiar groups, for a total of 17 samples, were included in the extended genetic typing of the 76 microhaplotypes (see Table S5).

The MPS results showed a good performance of the MH panel in the samples tested. The mean depth of coverage values ranged between 47.2 and 1,553 reads (median: 444.5; mean: 589.3). We observed uniformity of coverage greater than 90% for all samples (median: 92.9%; mean: 92.4%). Lower values of mean depth and reads on target were found for samples amplified with limited amounts of DNA (<0.1 ng).

Full MHs profiles were obtained for all buccal swabs (reference samples). Two bone remains (from Fam03 and Fam09) showed complete profiles, while three bone remains (from Fam05, Fam02, and Fam04) showed almost complete profiles with 2/76 missing loci. Finally, two skeletal remains (from Fam08 and Fam12) showed partial profiles with 12/76 MHs missing, likely due to the small amount (<0.1 ng) of degraded DNA used for amplification.

Family pedigrees were paired with the victims using the DVI module of the software Familias with settings adopted from the previous comparisons with the autosomal STR markers. Compared to the autosomal STR markers, the microhaplotype analyses were able to reach the 99.9% threshold for positive identification in two additional cases (Fam03 and Fam05) and increased the posterior probability value from 32.33% to 97.35% in Fam04. The MH and STR information can be combined if the markers are generically independent. In this case, MH and STR markers were more than 1 Mb apart [52], except two on chromosome 4, which are distanced by about 1 × 10^4^. We felt that the distance between the markers was sufficient to combine the information from the two sets of markers. Integrating the autosomal STRs and microhaplotypes in a combined likelihood ratio, a PP value (99.88%) close to the selected cut-off value was reached even for Fam04. In the remaining families, values lower than 20% were observed, thus suggesting inconclusive results. Among them, Fam12 showed a decrease in the posterior value, from 82.76% to 0.003% (0.16% combining STRs and microhaplotypes), suggesting a possible spurious match with the autosomal STRs only.

In conclusion, the kinship analyses provided posterior probabilities greater than 99.9% for six missing persons of six familial groups by considering 23 autosomal STRs (Fam01, Fam06, Fam07, and Fam10) and/or 76 microhaplotypes (Fam03 and Fam05). Among the remaining cases, Fam04 showed an increase in the likelihood ratio when the microhaplotypes were considered in the analysis and then combined with autosomal STR markers, thus reaching a posterior probability value close to the threshold (99.88%). In addition, the Y-chromosome markers supported the putative relationship. In three cases (Fam02, Fam08, and Fam09), no suggestion of a relationship was indicated by the autosomal STRs and microhaplotypes. Only haplotype compatibility between the post-mortem samples and the putative relatives with LR values equal to 1 × 10^4^ was detected, suggesting very strong support for a patrilineal relationship between the victim and the reference person. Moreover, the haplotypes were unique, thus strengthening the support for a putative paternal relationship. Finally, Fam12, which showed a PP > 80% by STR analysis, revealed a decrease in the LR and PP to very low values (0.003% and 0.16% by MH and STR + MH analyses).

### 3.7. Female DNA Profiles Comparisons

In order to investigate possible genetic relationships among the six female DNA profiles and between these profiles and the ones recovered from the male skeletal remains, a blind search approach was applied using the Familias DVI module. In particular, relationships up to the second degree (i.e., parent–child, full siblings, half-siblings) were considered. The results highlighted a putative full sibling relationship between two women (total number of shared alleles = 13/22, LR FS/NR = 3.24 × 10^2^ FS and NR stand for full siblings and non-relatives, respectively), while inconclusive results were obtained by pairing the female and male datasets.

## 4. Discussion

Personal identification through DNA typing is the gold standard in DVI scenarios. The successful typing of skeletal remains relies on employing an effective extraction method [14,16,51,53] and selecting the best performing skeletal elements [14,16,51,53]. In this study, DNA extraction was conducted from 179 bone/tooth powders using a semi-automated extraction protocol, which proved successful in minimizing human error and cross-contamination of samples, as well as in removing PCR inhibitors [31]. Also, the choice of the skeletal elements proved to be a decisive factor. Our results confirm that petrous bone outperforms other skeletal elements; in fact, DNA yields extracted from the inner part of this bone always gave values in the quantification range of the qPCR assays. In addition, although DNA degradation was a common feature of such aged samples, the overall quality of the STR profiles was rather high, with a limited incidence of PCR artefacts. As shown in Table 4, in fact, the percentages of allelic drop-out and allelic drop-in were no more than 3.1% and 0.9%, respectively.

As shown in Figure S1 and Figure S2, mainly the employment of petrous bones allowed us to implement the post-mortem database, highlighting the effectiveness of such bone elements in enhancing the overall success rate of molecular identification in forensic studies [31,32,33,51,53]. In our case, the anthropometric analysis established the minimum likely number of individuals (MLNI) [32] equal to 32; despite this, however, only a limited number of petrous bones (19 right and 19 left) were available for molecular analyses, yielding, in total, 24 unique genotypes. To increase the size of the post-mortem database, femurs, metacarpals, metatarsals, and teeth were used, with the short bones proving to be a promising, well-performing option [36,54,55]. The low rate of successful results from femurs is under investigation, but it is likely that the peculiar environmental conditions of that mass grave could have significantly impacted DNA preservation [38,56,57]. Overall, the conventional STR-CE approach allowed the identification of 30 consensus profiles (18 full and 12 partial). Among these 30 profiles, 6 were attributed to female individuals and were not used for the comparisons with AM data. A possible explanation for the discovery of these female individuals might be that they belonged to local women who had sentimental relationships with the missing Italian soldiers and/or were women fighting against Tito’s Communist Army, even if there are no records supporting these hypotheses.

The genetic profiles of the post-mortem database were then compared with those obtained from the putative relatives using the DVI module of the Familias software (Version 3.3.1). As recommended [42], the ante-mortem-driven approach was used in our calculations, considering the number of missing soldiers to be equal to 27. The definition of the number of victims in the ante-mortem proposition is a tricky issue because several factors need to be taken into account. In our case, the MLNI was 32; however, since 6 of them were females and the families were looking for the Italian soldiers who were supposedly buried in that area (*n* = 27), 27 seemed to be a conservative value. Lastly, the posterior probability for a positive identification was set to 99.9%, as suggested by several studies [13,42,47,48].

As shown in Table 5, the employment of these analytical parameters allowed the identification of four victims (three first-degree and a second-degree relationships) by considering 23 autosomal STRs. The distant kinship of the relatives was the main reason for the limited number of successful identifications as 2nd to 4th degree relationships were speculated from the genealogical data.

Microhaplotypes have the potential to be a valuable supplementary tool in complex kinship analysis given their specific advantages over traditional STR or SNP markers. In fact, these markers show advantages in typing highly degraded DNA samples, given the small size of the amplicons, and have the potential to investigate clan-and-extended family relationships [52]. In this study, a novel multiplex MPS panel containing 76 MHs was used as an additional tool to attempt to investigate up to 4th degree kinships. The amplicons in this panel were designed to be small (size lower than 140 bp), making them particularly advantageous for the analysis of these challenging skeletal remains. Overall, despite the small amplicon size, some samples showed partial profiles due to the small amount of DNA input used for library amplification. The results showed that the use of an MH panel allowed for improvement in the LR and posterior probability values in kinship analysis compared to the results obtained with STR loci alone. Moreover, even the single use of the MH panel allowed the identification of two additional victims (Fam03 and Fam05).

We decided to combine the two sets of markers to increase the information gained from the analyses conducted. We are confident that the 76 MHs are adequately spaced among the STR markers to be statistically independent for forensic analyses and statistical calculations [52]. Therefore, combining the informativity of STR and MH markers increased the likelihood ratio value of Fam04, resulting in a posterior probability value very close to the threshold (99.88%). The typing of the Y-chromosome markers supported the putative paternal relationship as well. 

While very low posterior probabilities for autosomal and MH markers were found for Fam02, Fam08, and Fam09 (combined PP < 15%), the analysis of Y-chromosome markers highlighted AM-PM haplotype sharing. Due to the different inheritance models and possible population substructure, we decided not to calculate a combined LR, considering both autosomal and Y-lineage markers. However, we considered the results obtained indicative of very strong support (LR = 10^4^) for a patrilineal relationship between the victim and the reference person. No match was obtained for Fam13 and Fam14 by lineage marker analysis.

Finally, Fam12, which showed a PP > 80% by STR analysis, showed a decrease in the LR and PP to very low values (0.003% and 0.16% by MH and STR + MH analyses), suggesting a possible spurious match with the STR markers. Therefore, for this family and for the last reference pedigree (Fam11), for which no match was obtained, further analyses will be performed in order to verify the presence/absence of the missing within the victim group.

## 5. Conclusions

This is the first report describing the identification of WWII Italian soldiers buried in a mass grave. Among the commingled remains, 24 male individuals were genotyped using conventional STR-CE and MPS molecular approaches. The comparisons with the reference samples belonging to 14 familiar groups representing the offspring of the missing Italian soldiers supported the identification, or the patrilineal relationship between the victim and the reference person, for 10 missing soldiers.

All the remains were transferred to the War Memorial of the Overseas Fallen Soldiers in Bari, Italy, in an official ceremony on 13 December 2024. The remains of four soldiers were then relocated and buried in the corresponding family vaults, according to the wishes of their relatives.

Furthermore, six female profiles were collected from the remains buried in the grave. Since no familial relationship up to the second degree was observed with the male group, they were assumed to be local women in a sentimental relationship with the missing Italian soldiers and/or were women fighting against Tito’s Communist Army. This investigation demonstrates the effectiveness of cooperation among geneticists, anthropologists, and historians in resolving challenging DVI cases at both forensic and humanitarian levels.

## Figures and Tables

**Table 1:** Bone/tooth samples analyzed in this study.

Skeletal Element	n
Right femur	28
Left femur	14
Right petrous bone	19
Left petrous bone	19
Metacarpal	19
Metatarsal	32
Molar tooth	16
Total	147

n: number of bone/tooth samples used for molecular analyses.

**Table 2:** Ante-mortem samples.

Kinship Degree	Relationship	Male	Female
1st Degree	Son/daughter	1	0
	Brother/sister	0	3
2nd Degree	Nephew/niece	5	6
	Grand-son/grand-daughter	1	0
3rd Degree	Half-nephew/half-niece	0	1
	Grand-nephew/Grand-niece	1	0
4th Degree	First cousin once removed	1	0

Relationship: relationship with the missing soldier. Male and female indicate the gender of the living relative.

**Table 3:** (**a**) Analyses performed by Quantifiler. (**b**) Analyses performed by PowerQuant.

a					
Skeletal Element	n	LOQ	>LOD	pg/µL	
Right femur	45	2.2%	26.7%	5 (14; *0*)	
Left femur	13	0%	46.2%	3 (5; *0*)	
Right petrous bone	19	100%	100%	440 (348; *402*)	
Left petrous bone	2	100%	100%	426 (356)	
Metacarpal	12	0%	83.3%	6 (7; *5*)	
Molar tooth	16	0%	31.3%	2 (3; *0*)	
Total	107	-	-	-	
**b**					
**Skeletal Element**	**n**	**LOQ**	**>LOD**	**pg/µL**	**DI**
Right femur	10	10.0%	90.0%	1 (1; *0*)	10.7 (n.a.)
Left femur	6	16.6%	100%	2 (2; *0*)	5.0 (n.a.)
Left petrous bone	17	100%	100%	362 (345; *329*)	28.6 (42; *11*)
Metacarpal	7	57.1%	100%	7 (6; *4*)	3.9 (2; *3*)
Metatarsal	32	81.3%	100%	37 (48; 14)	5.0 (2; *4*)
Total	72	-	-	-	-

(**a**,**b**). Quantification results. n: number of samples; LOQ: percentage of qPCR tests within the dynamic range of quantification (Limit of Quantification); >LOD: percentage of qPCR tests above the Limit of Detection; pg/μL: mean DNA concentrations (standard deviation and median value in the bracket; median value in italics); DI: mean degradation index (only for samples analyzed using the PowerQuant kit); and n.a.: not applicable.

**Table 4:** Stochastic phenomena assessed in autosomal STR analysis.

Skeletal Element	n	Markers	ADO	ADI
femur	19	18	17.7%	8.1%
petrous bone	57	19	3.1%	0.9%
metacarpal	13	18	10.9%	0.9%
metatarsal	32	15	4.9%	1.7%
molar tooth	1	n.a.	n.a.	n.a.
total	122	-	-	-

n: number of PCR tests yielding suitable profiles (full profiles and partial profiles); Markers: markers scored in partial profiles (median values); ADO: percentage of allelic drop-out phenomena; ADI: percentage of allelic drop-in phenomena; and n.a.: not applicable.

**Table 5:** Results of the pedigree analyses.

Fam ID	PP STRs (LR)	PP MHs (LR)	PP STRs + MHs (LR)	LR Y-STRs Western European	Pedigree
**Fam01**	99.99% (1.23 × 10^5^)				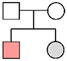
**Fam02**	2.52% (1.32)	11.66% (2.79)	14.94% (3.69)	1.24 × 10^4^	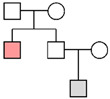
**Fam03**	96.98% (2.50 × 10^2^)	99.99% (6.23 × 10^5^)	>99.99% (1.56 × 10^8^)	2.20 × 10^1^	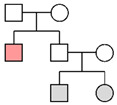
**Fam04**	32.33% (2.19 × 10^1^)	97.35% (7.73 × 10^2^)	99.88% (1.69 × 10^4^)	1.25 × 10^4^	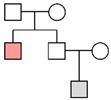
**Fam05**	99.59% (9.71 × 10^2^)	>99.99% (5.01 × 10^21^)	>99.99% (4.86 × 10^24^)		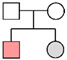
**Fam06**	>99.99% (2.00 × 10^7^)				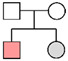
**Fam07**	99.93% (3.08 × 10^4^)				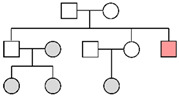
**Fam08**	4.45% (1.11)	6.37% (2.62)	6.20% (2.23)	1.07 × 10^4^	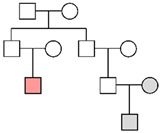
**Fam09**	1.73% (4.61 × 10^−1^)	0.19% (4.26 × 10^−2^)	0.09% (1.97 × 10^−2^)	1.15 × 10^4^	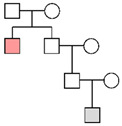
**Fam10**	>99.99% (9.31 × 10^7^)				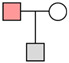
**Fam11**	9.76% (2.01)				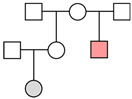
**Fam12**	82.76% (6.00 × 10^1^)	0.003% (5.57 × 10^−4^)	0.16% (3.34 × 10^−2^)		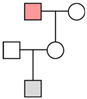
**Fam13**	1.19% (5.20 × 10^−2^)			No match	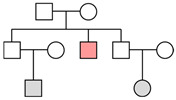
**Fam14**	8.71% (5.96 × 10^−1^)			No match	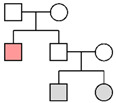

The results of the pedigree analyses (LR: likelihood ratio, PP: posterior probability, and MHs: microhaplotypes). In the last column, a graphic representation of the pedigrees is reported (colors represent the following persons: orange, victim; gray, reference persons; and white, relatives not available). Since for Y-chromosome STRs the order of magnitude was similar in the three reference populations, the most conservative LR value (corresponding to the Western European metapopulation) was reported. In cases of no matches with Y-STRs, the highest posterior probability value was considered for autosomal STR markers.

## Data Availability

Data are contained within the article or Supplementary Materials. Other data presented in this study and the genotyping data are available only upon reasonable request from the corresponding author, for genetic data protection issues.

## Supplementary Materials

The following supporting information can be downloaded at https://www.mdpi.com/article/10.3390/genes16030326/s1, Figure S1: Bone/tooth elements which contributed to the definition of each of the 30 genotypes; Figure S2: Implementation of the post-mortem database during the identification procedure (from February 2023 to May 2024); Table S1: List of the 147 samples used for DNA extraction; Table S2: Samples used for autosomal STR typing; Table S3: Main features of the six female genotypes; Table S4: Description of the 14 pedigrees analyzed in this study; and Table S5: Samples analyzed with MHs.
