# Supplementary material for: Molecular Identification of the Italian Soldiers Found in the Second World War Mass Grave of Ossero
Source: Genes (Basel). 2025 Mar 11;16(3):326. doi: 10.3390/genes16030326 (PMC11942473; doi:10.3390/genes16030326)
Supplement: Supplementary file 1 [file genes-16-00326-s001.zip › genes-3452519-supplementary.pdf]

Supplementary Materials

Table S1. List of the 147 samples used for DNA extractions (n= 179)

Skeletal element	n	Extracted once	Extracted twice	Extracted 3 times	Total	Quantifiler	PowerQuant
Right femur	28	8	13	7	55	45	10
Left femur	14	9	5	0	19	13	6
Right petrous bone	19	19	0	0	19	19	0
Left petrous bone	19	19	0	0	19	2	17
Metacarpal	19	19	0	0	19	12	7
Metatarsal	32	32	0	0	32	0	32
Molar tooth	16	16	0	0	16	16	0
Total	147	122	18	7	179	107	72

Quantifiler and PowerQuant columns indicate the number of qPCR tests performed with those kits.

Table S2. List of the 126 samples used for autosomal STR typing

Skeletal element	>LOD	STR tests	post-mortem profiles	new genotypes
Femur	33	39	13	6
Petrous bone	38	57	38	19
Metacarpal	18	20	11	2
Metatarsal	32	35	29	3
Molar tooth	5	9	1	0
Total	126	160	92	30

>LOD: number of samples > Limit of Detection in at least one qPCR test; STR tests: number of PCR-STR tests; Post-mortem profiles: number of STR profiles yielded from each bone element; New genotypes: number of new genotypes identified through each bone element.

Table S3. Main features of the six female genotypes

Genotype	Bone elements	STR
#18	3	16/17
#19	2	17/17
#20	2	16/17
#22	1	15/17
#27	2	17/17
#28	1	12/17
Total	11	/

Genotype: genotype code; bone elements: number of bone elements which showed the same female genotype; STR: number of autosomal markers typed.

Table S4. Description of the 14 pedigrees analyzed in this study

Fam ID	Reference	Missing	Degree	Pedigree
Fam01	OR1 (Sister)	Brother	1	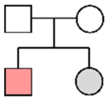
Fam02	OR2 (Nephew)	Uncle	2	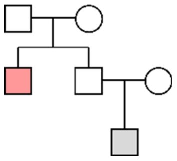
Fam03	OR3 (Nephew)	Uncle	2	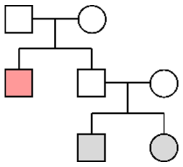
	OR4 (Niece)	Uncle	2	
Fam04	OR5 (Nephew)	Uncle	2	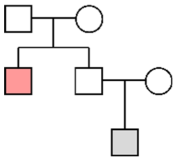
Fam05	OR6 (Sister)	Brother	1	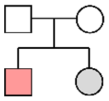
Fam06	OR7 (Sister)	Brother	1	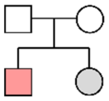
Fam07	OR8 (Niece)	Uncle	2	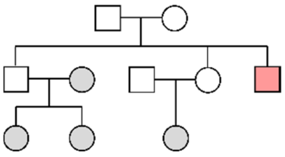
	OR9 (Niece)	Uncle	2	
	OR10 (Sister-in-law)	Brother-in-law	-	
	OR11 (Niece)	Uncle	2	

Fam08	OR12 (First Cousin once removed)	First Cousin once removed	4	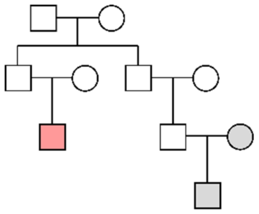
	OR13 (Mother of OR12)	-	-	
Fam09	OR14 (Grandnephew)	Great-uncle	3	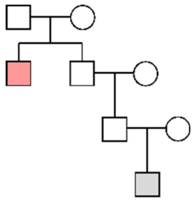
Fam10	OR15 (Son)	Father	1	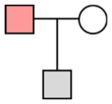
Fam11	OR16 (Half-niece)	Half-uncle	3	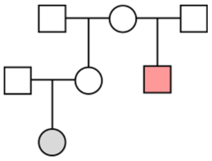
Fam12	OR18 (Grandson)	Grandfather	2	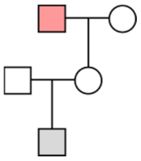
Fam13	OR19 (Niece)	Uncle	2	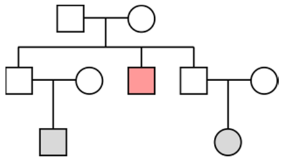
	OR20 (Nephew)	Uncle	2	
Fam14	OR22 (Nephew)	Uncle	2	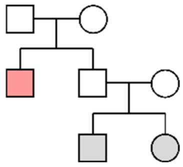
	OR23 (Niece)	Uncle	2	

The colours represent the following persons: orange, missing soldier; grey, reference persons (living donor); and white, relatives not available.

Table S5. Samples analyzed with MHs

Family	Sample	Input	pM	M.R.	O.T.%	M.D.C.	unif.	Typed
Fam02	OR2	0.37	134.4	50,349	93.1	590.7	96.5 %	76/76
	XR12	0.80	552.7	16,012	45.5	91.71	96.0 %	74/76
Fam03	OR3	0.94	951.4	65,810	92.6	770.9	92.2 %	76/76
	OR4.2	1	457.9	105,466	95.7	1278	92.9 %	76/76
	XR6	0.94	497.4	82,053	86.2	892.3	93.1 %	76/76
Fam04	OR5	1	434.3	117,096	96.9	1453	91.0 %	76/76
	XM20	0.12	717.4	31,776	18.6	74.93	92.8 %	71/76
	XM20	0.12	2763.4	45,949	27.4	156.3	94.0 %	74/76
Fam05	OR6	0.66	342.1	125,924	96.5	1553	90.0 %	76/76
	XR8	0.16	650.2	28,103	38.7	134.9	93.2 %	72/76
	XR8	0.16	2647.5	35,366	36.7	160.2	93.5 %	74/76
Fam08	OR12	1	171.5	33,054	93.0	387.8	95.6 %	76/76
	XM16	0.09	411.8	11,099	33.6	47.21	89.5 %	64/76
Fam09	OR14	1	378.3	96,608	92.8	1138	93.2 %	76/76
	XR11	0.48	765.0	51,080	68.8	444.5	92.7 %	76/76
Fam12	OR18.2	0.90	35.9	78,693	84.4	726.2	92.9 %	76/76
	XR17	0.1	84.3	14,801	65.8	119.1	81.0 %	64/76

Input: template DNA used for PCR amplification; pM: quantification of the libraries (in picomoles); M.R.: mapped reads; O.T.%: on target percentage; M.D.C.; mean depth of coverage; unif.: uniformity; Typed: number of typed MHs.

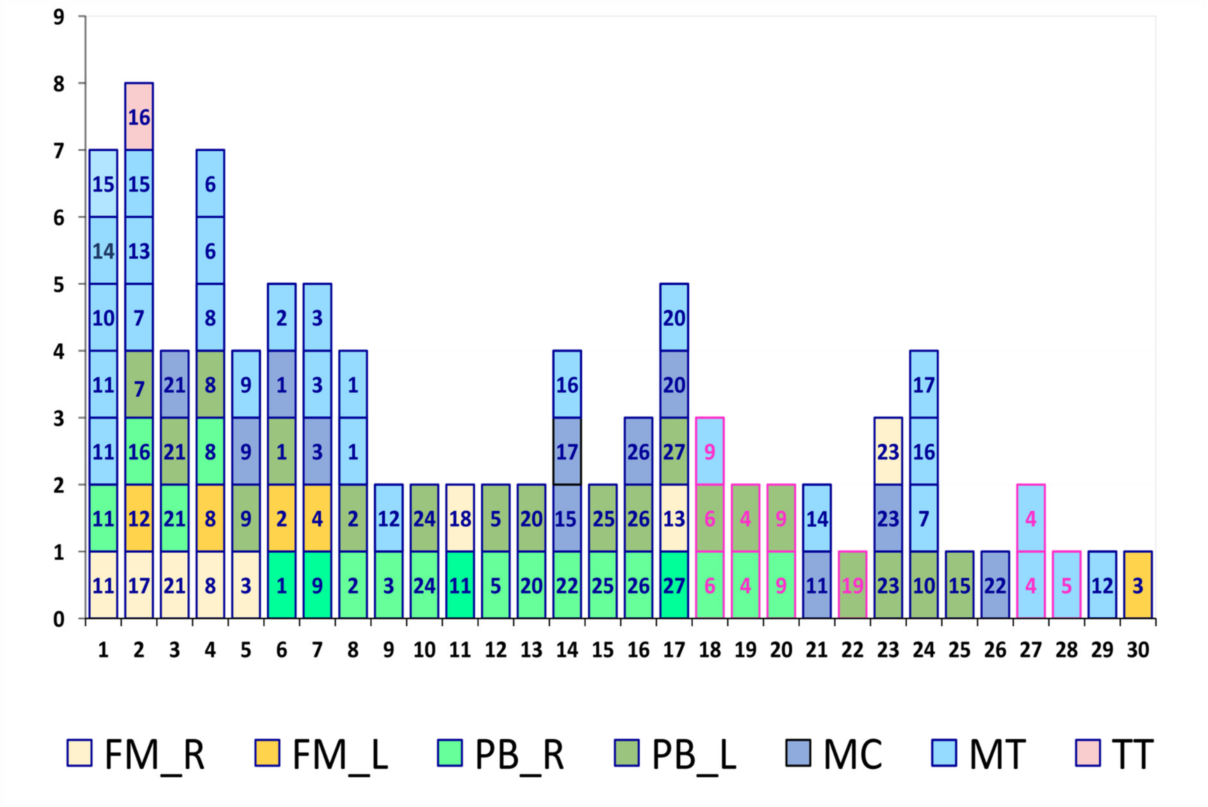

Figure S1. Ninety-two samples which contributed to the definition of each of the 30 genotypes. X-axis: genotypes #1 - #30 (in chronological order of identification); Y-axis: number of skeletal elements which contributed to the definition of each genotype. Each box represents a single bone/tooth element, with the different colours indicating different elements. The number inside each box corresponds to the metal casket in which the element was found. FM_R: right femur; FM_L: left femur; PB_R: right petrous bone; PB_L: left petrous bone; MC: metacarpal; MT: metatarsal; TT: molar tooth. The six female genotypes are indicated in pink colour. As reported in Table A2, these 30 unique consensus genotypes were yielded from 122 PCR analyses which showed at least 12 STR markers.

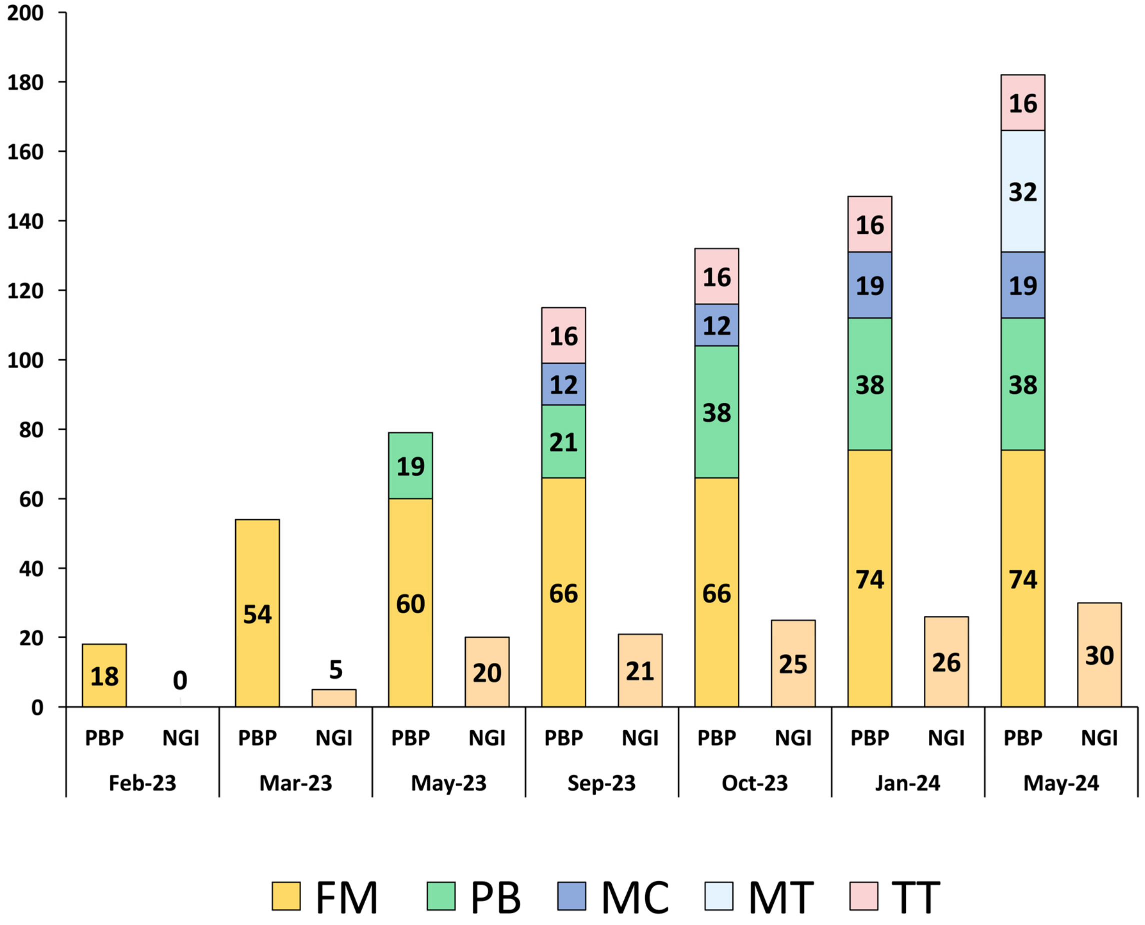

Figure S2. Implementation of the post-mortem database during the identification procedure (from February 2023 to May 2024). The X-axis shows the processed bone/tooth powders (PBP) and new genotypes identified (NGI), respectively. Y-axis: number of PBP and, respectively, NGI. The different skeletal elements are shown in different colors, as specified in the legend (FM: femur; PB: petrous bone; MC: metacarpal; MT: metatarsal; TT: tooth). The numbers inside the bars refer to the progressive values of the processed bone/tooth powders and, respectively, the new genotypes identified.
